# Development of an emergency department triage tool to predict admission or discharge for older adults

**DOI:** 10.1186/s12245-025-00825-3

**Published:** 2025-02-14

**Authors:** Ashraf Abugroun, Saria Awadalla, Sanjay Singh, Margaret C. Fang

**Affiliations:** 1 Division of Hospital Medicine, University of California, 505 Parnassus Ave, San Francisco, CA 94143 USA; 2 Division of Biostatistics, University of Illinois Chicago, Chicago, IL USA; 3 Department of Medicine, Medical College of Wisconsin, Milwaukee, WI USA

**Keywords:** Hospitalization, Emergency department, Risk score, Older adults

## Abstract

**Background:**

Older adults present to Emergency Departments (ED) with complex conditions, requiring triage models that support effective disposition decisions. While existing models perform well in the general population, they often fall short for older patients. This study introduces a triage model aimed at improving early risk stratification and disposition planning in this population.

**Methods:**

We analyzed the National Hospital Ambulatory Medical Care Survey data (2015–2019) for ED patients aged ≥ 60 years, excluding those who died in the ED or left against medical advice. Key predictors were identified using a two-step process combining LASSO and backward stepwise selection. Model performance was evaluated using AUC and calibration plots, while clinical utility was assessed through decision curve analysis. Risk thresholds (< 0.1, 0.1–0.5, > 0.5) stratified patients into low, moderate, and high-risk groups, optimizing the balance between sensitivity and specificity.

**Results:**

Of 13,431 patients, 3,180 (23.7%) were admitted. Key predictors for admission included ambulance arrival, chronic conditions, gastrointestinal bleeding, and abnormal vital signs. The model showed strong discrimination (AUC 0.73) and good calibration, validated by 10-fold cross-validation (mean AUC 0.73, SD 0.02). Decision curve analysis highlighted net benefit across clinically relevant thresholds. At thresholds of 0.1 and 0.5, the model identified 18.9% as low-risk (91.2% accuracy) and 7.9% as high-risk (57.7%). Adjusting thresholds to 0.2 and 0.4 expanded low-risk (55.4%, 87.9% accuracy) and high-risk (14.1%, 53.7% accuracy) groups.

**Conclusions:**

This older adult–focused risk score uses readily available data to enhance early discharge, prioritize admissions for high-risk patients, and enhance ED care delivery.

**Supplementary Information:**

The online version contains supplementary material available at 10.1186/s12245-025-00825-3.

## Highlights

Readily available triage data predict hospital admission in older adult ED patients.

Key predictors include chief complaint, ambulance arrival, comorbidities, and vital signs.

The Hospital Admission Model effectively stratifies patients into low- and high-risk groups.

At a 0.2 threshold, 55% of patients were classified as low risk with 88% accuracy.

At a 0.5 threshold, 8% of patients were classified as high risk with 58% accuracy.

**Supplementary Information:**

The online version contains supplementary material available at 10.1186/s12245-025-00825-3.

## Introduction

Emergency department (ED) visits in the United States have increased significantly, with adults aged 60 and older now accounting for 20–24% of all visits [1–3]. Older adults frequently present with complex, multifactorial conditions that necessitate timely and carefully considered disposition decisions [4–8]. Predictive models are increasingly used at triage to identify patients at high risk for hospital admission, thereby guiding resource allocation and balancing wait times [9, 10]. However, most existing models focus solely on predicting admission, overlooking the equally critical need to recognize low-risk patients who may be safely discharged [11, 12]. Additionally, many existing models rely on non-triage data, such as laboratory or imaging results, making them less practical for early decision-making. Few models have been validated in older adults, and reliance on single-institution datasets further restricts their generalizability [10, 13–16]. Compounding these issues, attempts to incorporate frailty measures into ED triage assessments have had limited success, offering minimal guidance when prompt decisions are required [17, 18]. In response to these gaps, this study develops and validates an older adult -focused risk prediction model that leverages readily available triage data to predict both admission and discharge outcomes. By enabling early risk stratification, this tool aims to strengthen clinical decision-making, support timely dispositions, and ultimately improve ED care for older adults.

## Methods

Study protocols and results were reported following the Transparent Reporting of a Multivariable Prediction Model for Individual Prognosis or Diagnosis (TRIPOD) guidelines for cohort studies (Supp. Table 1). This study utilized publicly available data from the National Hospital Ambulatory Medical Care Survey (NHAMCS). As the data were deidentified and publicly accessible, institutional review board approval was not required.

### Data source

Data for this retrospective study were drawn from the NHAMCS for the years 2015–2019 [19]. The NHAMCS is a publicly available database collected by the U.S. Census Bureau on behalf of the National Center for Health Statistics, a division of the Centers for Disease Control and Prevention. The survey, conducted annually since 1992, gathers data on ambulatory care in U.S. hospital emergency and outpatient departments. The NHAMCS employs a three-stage probability sampling design to select visits from non-federal, general, and short-stay hospitals across all states and the District of Columbia, excluding federal, military, and Veterans Administration hospitals. This design involves sampling geographical areas, hospitals within these areas, and ultimately, emergency service areas within the selected hospitals. Data is collected through interviews conducted by Census interviewers using a computerized Patient Record Form during a designated 4-week reporting period. The collected data includes patient demographics, reasons for visits, diagnoses, services provided, and characteristics of the facilities.

### Study population and outcome

This study included older patients aged 60 years or older who visited the Emergency Department (ED) (Supp. Figure 1a). Age 60 years and above was chosen to align with World Health Organization definitions, ensuring broader applicability [20]. The primary outcome was admission to inpatient care, including direct admissions and those following an observation stay. Of 75,948 patients, 13,431 met the inclusion criteria. Exclusions were made for age younger than 60 years (*n* = 59,495), leaving the ED before treatment completion (*n* = 304), ED death (*n* = 64), no documented chief complaint (*n* = 57), incomplete initial vital signs (*n* = 2,188) or unrecorded mode of arrival (*n* = 547). With 13,431 participants, the study had > 99% power to detect odds ratios as small as 1.1 (α = 0.05) (Supp. Figure 1b), ensuring robust statistical power.

### Candidate predictor variables

This study investigated predictors of hospital admission from the emergency room. We identified potential predictors from established frameworks and prior research (Supp. Table 2), ensuring the validity and comparability of our findings [9]. Table 1 summarizes the variables included in our analysis, encompassing patient demographics, emergency department visit characteristics, hospital factors, clinical factors (comorbidities and presenting symptoms), and triage vital signs, which were dichotomized based on established clinical thresholds.

### Statistical analysis

Continuous variables were summarized as medians with interquartile ranges, and categorical variables as frequencies. We excluded individuals with missing data on key admission variables (vital signs and mode of arrival), representing 3–7% of the sample. Missingness in all other variables, was addressed through multiple imputation using the ‘mice’ package. Covariate balance was assessed using standardized mean differences. (Supp. Figure 2) [21]. An effect size of 0.1 or greater indicated a significant covariate imbalance between groups. A two-step variable selection process was employed for model building, using hospital admission as the primary outcome [22]. The dataset was divided into a 70% training set and a 30% testing set for model development and validation. Initially, 41 out of 65 predictors were selected based on clinical relevance. The least absolute shrinkage and selection operator (LASSO) regression was utilized to identify key predictors using the ‘glmnet’ package, selecting the lambda that minimized mean-squared error via tenfold cross-validation, based on the ‘one standard error’ rule to ensure model parsimony (Suppl. Figures 3–6) [23]. Subsequently, the variables were refined using backward stepwise selection according to the Akaike information criterion (AIC), which enhanced model parsimony, face validity, and reduced collinearity. This adjustment improved the Bayesian Information Criterion (BIC) and only slightly altered the area under the receiver operating characteristic curve (AUC) [24]. Survey weights were not applied, as the primary goal was to develop a practical risk prediction model rather than achieve national representativeness. The final model underwent internal validation with 10-fold cross-validation to ensure stability and generalizability using the ‘caret’ package and was then tested on an independent testing cohort.

**Fig. 1:**
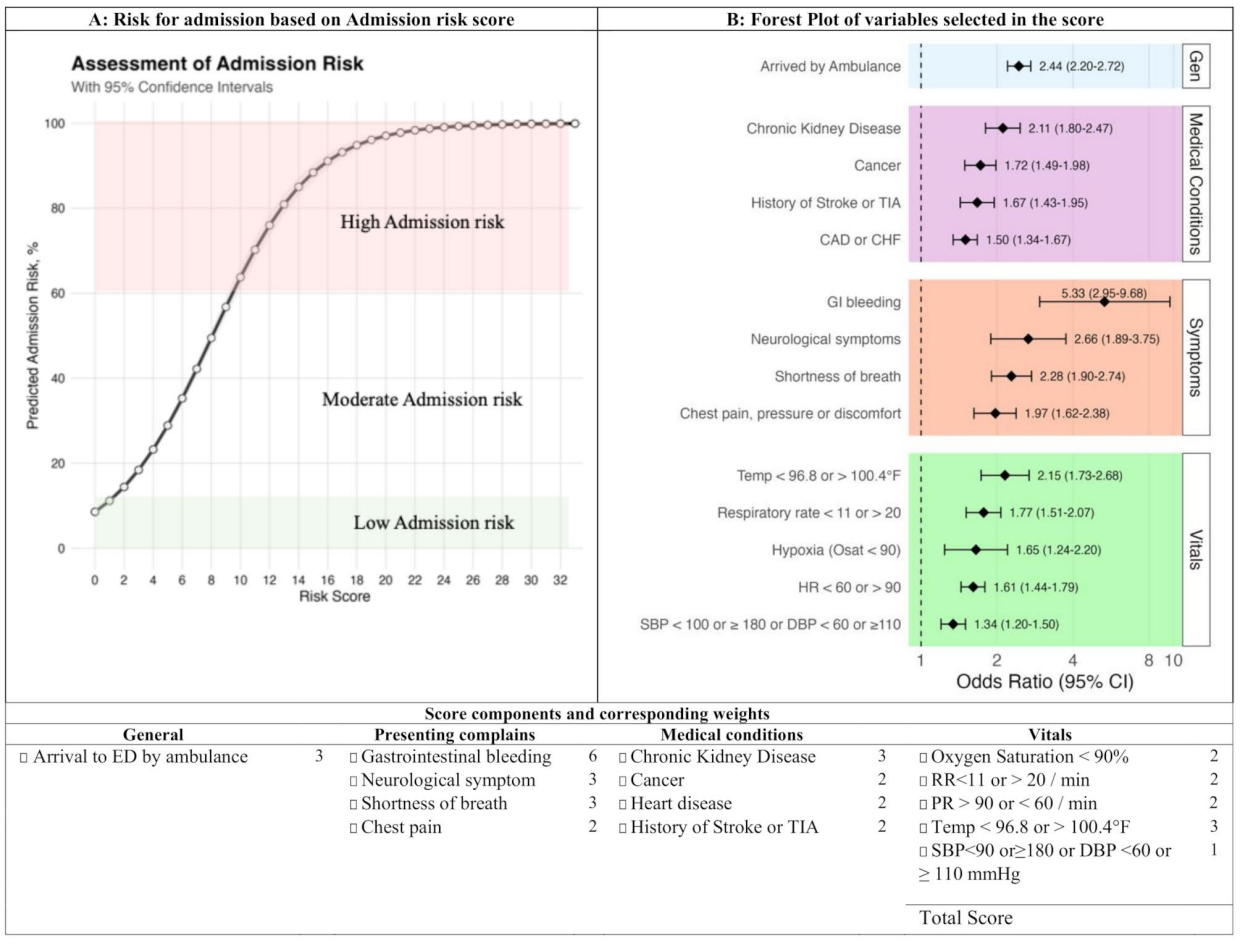
Risk for admission score estimator. (**A**) This plot shows the association between hospital admission risk score and predicted probability for admission. (**B**) Forest plot shows odds ratios (OR) with 95% confidence intervals for factors associated with hospital admission, categorized into General, Medical Conditions, Symptoms, and Vitals. The dashed vertical line at OR = 1.0 indicates no association with admission

From the final logistic regression model, we developed a risk score by identifying the smallest positive coefficient as the reference value and dividing all nonzero coefficients by this value to obtain relative weights. These weights were rounded to integers to create an additive scoring system. The resulting risk score estimates individual hospital admission probability (Fig. 1, Supp. Tables 3 and Supp. Figure 7). Additionally, A nomogram was developed using the ‘rms’ package to provide a visual representation of the prediction model. (Supp. Figure 8). The sensitivity, specificity, of the receiver operating characteristic (ROC) curve were plotted to assess the model’s performance using the ‘pROC’ package. Calibration was tested by comparing the predicted probabilities with the observed outcomes using a calibration curve. A decision curve analysis was performed using the ‘rmda’ package to evaluate the clinical utility of the admission risk score. This analysis assessed the net benefit of the risk score across a range of risk thresholds compared to the strategies of admitting all patients or admitting no patients [25]. Based on the predicted probability for admission derived from the final model, we defined three risk groups: low (< 0.1), moderate (0.1 to 0.5), and high (> 0.5). These cut points were determined using model performance data, including the balancing of sensitivity, specificity, and insights from the decision curve analysis. All analyses were conducted using R version 4.3.2 (2023-10-31), with a significance threshold set at 0.05.

### Sensitivity analysis

Initial Emergency Department vital signs (temperature, blood pressure, heart rate, respiratory rate, oxygen saturation) and mode of arrival were identified as essential disposition predictors. These key variables had 3–7% incomplete records (Supplementary Fig. 1). We compared two analytical approaches: multiple imputation (MI), which included all eligible patients (*n* = 16,028), and complete case analysis (CCA), which included only patients with fully documented variables (*n* = 13,431).

## Results

### Study population characteristics

Of the 13,431 patients presenting to the ED during the study period, 3,180 (23.7%) were admitted (Table 1). The median wait time to see a provider was 18 min (IQR: 6 to 35), with 13.3% of patients experiencing waits exceeding one hour. Among participating hospitals, 77.7% reported instances of admitted patients experiencing ED boarding times exceeding two hours. Compared to those not admitted, admitted patients were older (median age 74 vs. 71 years; SMD, 0.28), more likely to be nursing home residents and Medicare beneficiaries, and more frequently arrived by ambulance. They also exhibited a higher prevalence of chronic conditions. The most common admission complaints were shortness of breath, chest pain, and neurological symptoms, often accompanied by abnormal vital signs.

**Table 1:** Baseline characteristics of the study population by admission status

Label	Overall	Admit to Hospital		SMD
		No	Yes	
*n*	13,431	10,251	3180	
Male sex	5884 (43.8)	4433 (43.2)	1451 (45.6)	0.05
Age in years	72.0 [65.0, 81.0]	71.0 [65.0, 80.0]	74.0 [67.0, 83.0]	0.28
**Race and Ethnicity**				0.09
Non-Hispanic White	9607 (71.5)	7275 (71.0)	2332 (73.3)	
Non-Hispanic Black	2136 (15.9)	1692 (16.5)	444 (14.0)	
Hispanic	1110 (8.3)	866 (8.4)	244 (7.7)	
Non-Hispanic Other	578 (4.3)	418 (4.1)	160 (5.0)	
Nursing home resident	720 (5.4)	442 (4.3)	278 (8.7)	0.18
Initial Emergency Department visit	804 (6.0)	607 (5.9)	197 (6.2)	0.01
Arrived by Ambulance	3943 (29.4)	2432 (23.7)	1511 (47.5)	0.51
Weekend Admission	3550 (26.4)	2773 (27.1)	777 (24.4)	0.06
**Time of Emergency Department visit**				0.04
7:00 AM-7:00 PM	10,293 (76.6)	7865 (76.7)	2428 (76.4)	
8:00 PM- 1:00 AM	2186 (16.3)	1681 (16.4)	505 (15.9)	
2:00 AM-6:00 AM	952 (7.1)	705 (6.9)	247 (7.8)	
**Season**				0.05
Winter	3355 (25.0)	2585 (25.2)	770 (24.2)	
Spring	3629 (27.0)	2735 (26.7)	894 (28.1)	
Summer	3125 (23.3)	2356 (23.0)	769 (24.2)	
Autumn	3322 (24.7)	2575 (25.1)	747 (23.5)	
**Insurance Type**				
Medicare insurance	9094 (67.7)	6738 (65.7)	2356 (74.1)	0.18
Private insurance	5318 (39.6)	4052 (39.5)	1266 (39.8)	0.01
Medicaid insurance	2453 (18.3)	1922 (18.7)	531 (16.7)	0.05
Emergency Department Residency Program present	3644 (27.1)	2730 (26.6)	914 (28.7)	0.05
Emergency Department bed Coordinator present	10,188 (75.9)	7588 (74.0)	2600 (81.8)	0.19
Admitted patients ever boarded > 2 h.	10,432 (77.7)	7785 (75.9)	2647 (83.2)	0.18
Wait time in Emergency Department	18.0 [6.0, 35.2]	18.0 [7.0, 35.2]	16.0 [6.0, 35.2]	0.01
Wait time before first provider ≥ 1 h	1780 (13.3)	1375 (13.4)	405 (12.7)	0.02
**Chronic conditions**				
History of Pulmonary Embolism	517 (3.8)	338 (3.3)	179 (5.6)	0.11
History of Heart disease	3659 (27.2)	2382 (23.2)	1277 (40.2)	0.37
History of Alzheimer’s disease/Dementia	788 (5.9)	506 (4.9)	282 (8.9)	0.16
History of Asthma	1116 (8.3)	829 (8.1)	287 (9.0)	0.03
History of Cancer	1612 (12.0)	1076 (10.5)	536 (16.9)	0.19
History of Stroke or Transient Ischemic Attack	1371 (10.2)	871 (8.5)	500 (15.7)	0.22
History of Chronic Kidney Disease	1246 (9.3)	717 (7.0)	529 (16.6)	0.30
History of Chronic Obstructive Pulmonary Disease	2114 (15.7)	1391 (13.6)	723 (22.7)	0.24
History of Depression	1785 (13.3)	1312 (12.8)	473 (14.9)	0.06
History of End stage renal disease	288 (2.1)	178 (1.7)	110 (3.5)	0.11
Obesity ( Body Mass Index > 30)	944 (7.0)	622 (6.1)	322 (10.1)	0.15
History of Obstructive Sleep Apnea	750 (5.6)	528 (5.2)	222 (7.0)	0.08
Substance abuse or dependence	517 (3.8)	382 (3.7)	135 (4.2)	0.03
Alcohol misuse, abuse, or dependence	418 (3.1)	300 (2.9)	118 (3.7)	0.04
History of Diabetes Mellitus	3778 (28.1)	2725 (26.6)	1053 (33.1)	0.14
Number of chronic conditions	2.0 [1.0, 4.0]	2.0 [1.0, 3.0]	3.0 [2.0, 5.0]	0.50
**Presenting condition**				
Fracture or dislocation	36 (0.3)	26 (0.3)	10 (0.3)	0.01
Motor Vehicle Accident	66 (0.5)	63 (0.6)	3 (0.1)	0.09
Accident including falls	440 (3.3)	363 (3.5)	77 (2.4)	0.07
Back pain	1329 (9.9)	1196 (11.7)	133 (4.2)	0.28
Chest pain, pressure or discomfort	895 (6.7)	601 (5.9)	294 (9.2)	0.13
Shortness of breath	964 (7.2)	500 (4.9)	464 (14.6)	0.33
Neurological symptoms	218 (1.6)	122 (1.2)	96 (3.0)	0.13
Gastrointestinal bleeding	66 (0.5)	35 (0.3)	31 (1.0)	0.08
**Abnormal Vitals**				
Temp < 96.8 or > 100.4 °F	619 (4.6)	352 (3.4)	267 (8.4)	0.21
SBP < 100 or ≥ 180 or DBP < 60 or ≥ 110 mmHg	3357 (25.0)	2383 (23.2)	974 (30.6)	0.17
HR < 60 or > 90 beats/min	4527 (33.7)	3149 (30.7)	1378 (43.3)	0.26
Respiratory rate < 11 or > 20 breaths/min	1406 (10.5)	797 (7.8)	609 (19.2)	0.34
Hypoxia (O_2_sat < 90%)	361 (2.7)	180 (1.8)	181 (5.7)	0.21
Pain scale > 7	2447 (18.2)	2003 (19.5)	444 (14.0)	0.15

N: Sample size, SMD: Standardized mean difference, SBP: Systolic Blood Pressure; DBP: Diastolic Blood Pressure

### Risk score and model performance

**Fig. 2:**
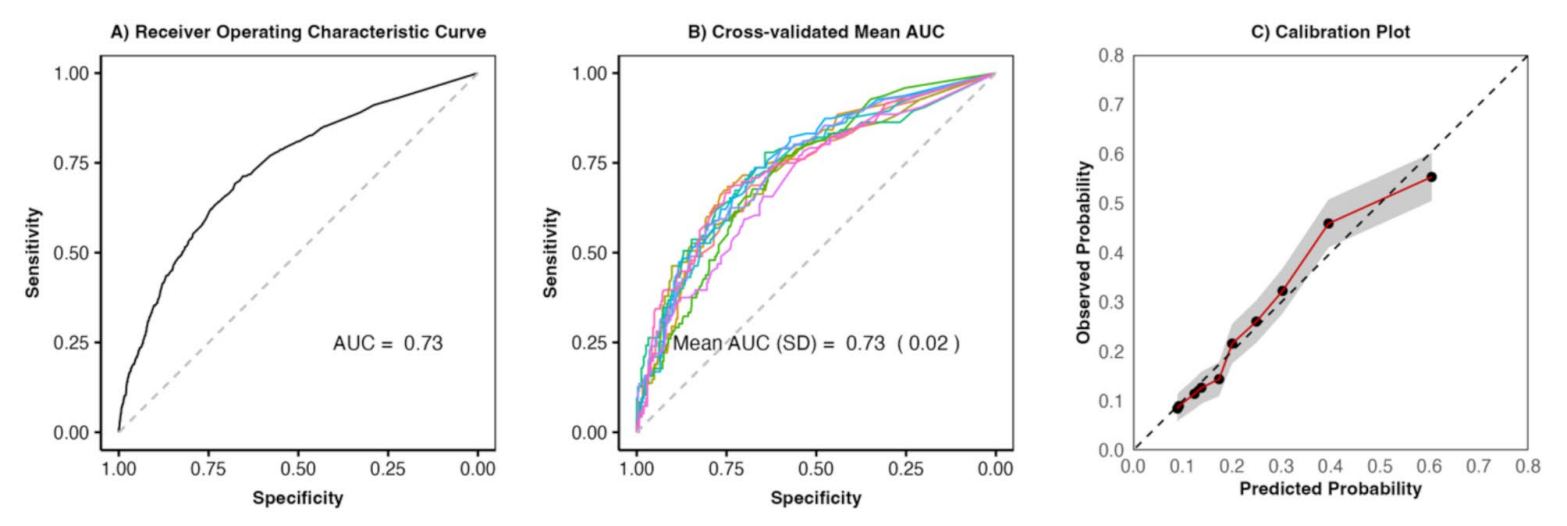
Model performance. (**A**) Receiver operating characteristics (ROC) curve showing the relationship between sensitivity (Y-axis) and 1-specificity (X-axis) in determining the ability of hospital admission risk score in predicting admission. The area under the ROC curve (AUC) for the score is 0.73. (**B**) The Cross-validated (cv) mean AUC is 0.73, SD = 0.02. (**C**) Calibration plot of expected to observed risk of admission. The 45-degree bisector associated with the identity between predicted probabilities and observed responses. Shaded area represents the 95% CI

Key predictors included in the admission risk score were ambulance arrival, gastrointestinal bleeding, neurological symptoms, chronic conditions, and abnormal vital signs (Fig. 1). The hospital admission risk prediction model achieved an accuracy of 68% (95% CI, 67–69%), with sensitivity of 0.69, specificity of 0.68, positive predictive value of 0.40, and negative predictive value of 0.88. Figure 2 illustrates the model’s performance, showing an AUC of 0.73, indicating good discrimination between admitted and non-admitted patients. The K-fold cross-validation confirmed model robustness with a mean AUC of 0.73 (SD, 0.02), and calibration plots demonstrated strong alignment between predicted and observed probabilities across all deciles, with minor discrepancies in the extremes (Supp. Table 4).

### Defining risk thresholds for hospital admission prediction

**Fig. 3:**
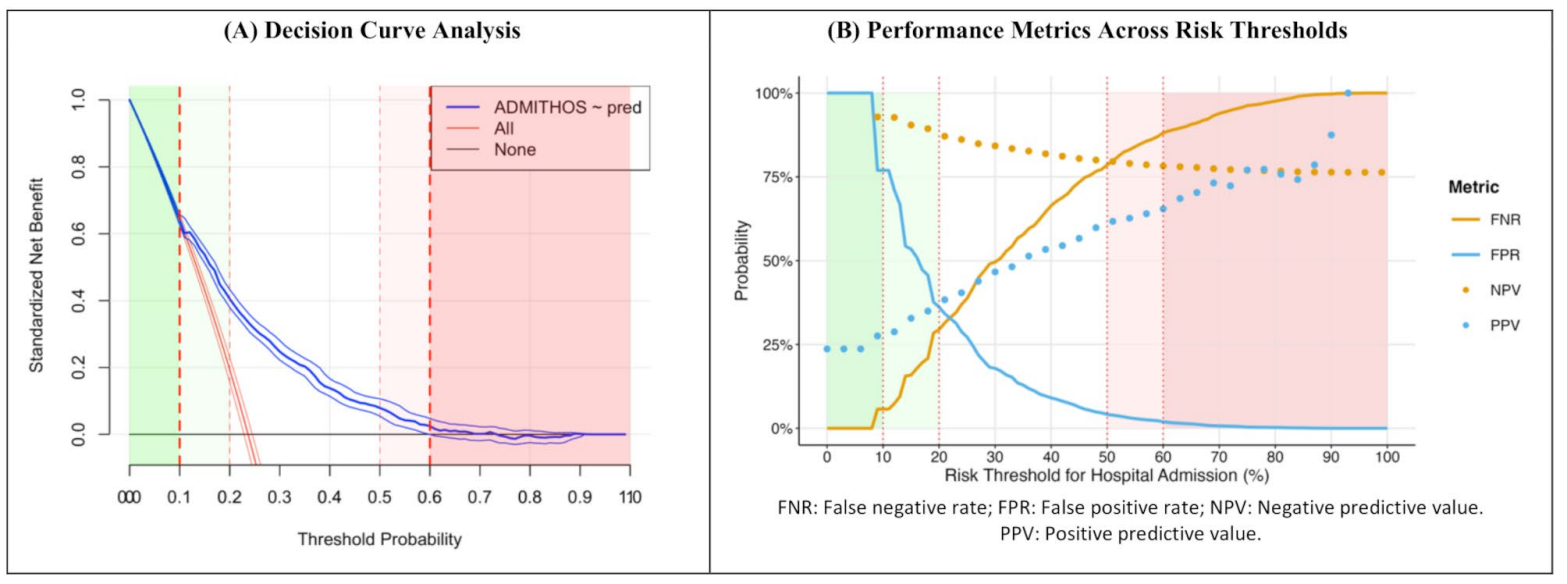
Defining risk groups using decision curve analysis and performance metrics across admission probability thresholds. (**A**) The Decision Curve Analysis (DCA) plot evaluates the clinical usefulness of a predictive model. The X-axis shows the threshold probability for taking action, while the Y-axis represents the standardized net benefit. The blue curve represents the model’s net benefit across different thresholds, compared to the red lines indicating net benefits if everyone (solid red) or no one (dashed red) were treated. The green shaded region highlights thresholds where the model provides a positive net benefit, while the red region shows where the benefit decreases. (**B**) This graph shows the relationship between diagnostic metrics (FNR, FPR, NPV, PPV) and risk thresholds for hospital admission. As the threshold increases, the False Negative Rate (FNR) incasese and False Positive Rate (FPR) decrease, while Positive Predictive Value (PPV) increases and Negative Predictive Value (NPV) decreases. The green and red shaded areas highlight threshold ranges where these metrics are optimized for clinical decision-making

**Fig. 4:**
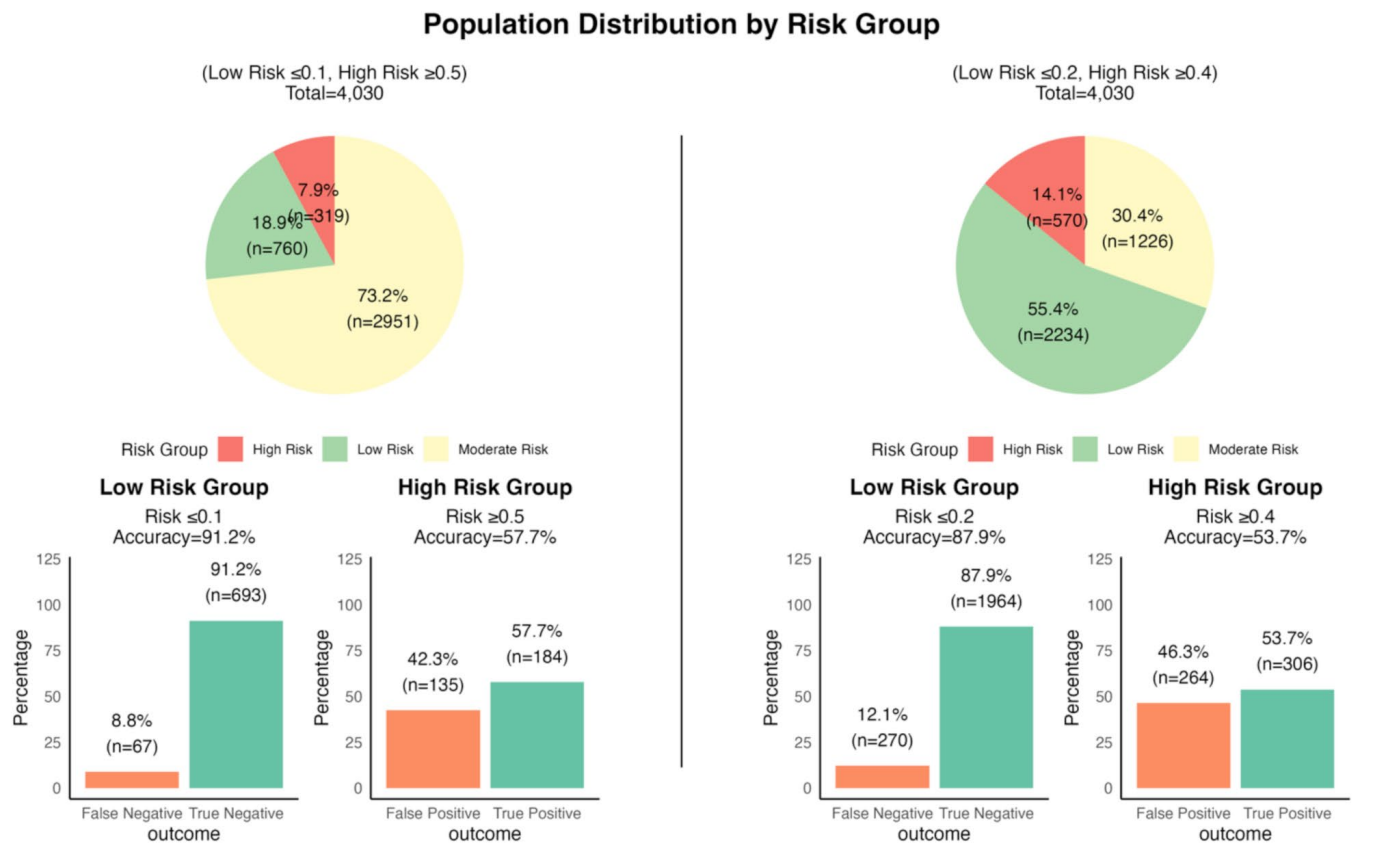
Application of selected probability thresholds on test database. This figure shows population distributions by risk group with pie charts and performance metrics for two risk threshold models. The left panel uses thresholds of ≤ 0.1 for low risk and ≥ 0.5 for high risk, while the right panel uses ≤ 0.2 for low risk and ≥ 0.4 for high risk. Bar charts display accuracy, true negatives, and false positives for each group

To facilitate clinical interpretation, the risk score was used to calculate a predicted probability of admission for each individual, stratifying patients into three risk groups: low, moderate, and high. Thresholds for these groups were determined based on model performance metrics, including sensitivity, specificity, and decision curve analysis. The model demonstrated its greatest clinical utility at lower thresholds, effectively identifying low-risk patients with high Negative Predictive Value (NPV) and minimizing false negatives (Fig. 3a). As thresholds increased, the ability to correctly identify high-risk patients improved, but at the cost of misclassifying some true positives. This trade-off is illustrated in (Fig. 3b), where lower thresholds highlight the model’s high NPV and low false negative rate, while higher thresholds show increased Positive Predictive Value (PPV) and reduced false positive rate. Based on these findings, thresholds of 0.1 for low risk and 0.5 for high risk were chosen to achieve an optimal balance between minimizing false negatives and reducing false positives. At these thresholds, the model classified 18.9% of patients as low risk (91.2% accuracy) and 7.9% as high risk (57.7% accuracy), with the remaining 73.2% in the moderate-risk group (Fig. 4a). To explore a more inclusive approach, thresholds were adjusted to 0.2 for low risk and 0.4 for high risk. This resulted in 55.4% of patients being classified as low risk (87.9% accuracy) and 14.1% as high risk (53.7% accuracy) (Fig. 4b). While this approach captured more patients at both extremes of risk, it reduced overall accuracy, particularly in the high-risk group, underscoring the trade-off between sensitivity and specificity.

### Sensitivity analysis

Both multiple imputation (MI) and Complete case analysis (CCA) models showed identical discrimination (AUC 0.73). The MI model selected 16 variables while CCA identified 14 variables, with comparable effect sizes for key predictors (e.g., GI bleeding: OR 4.6 vs. 5.33; arrived by ambulance: OR 2.55 vs. 2.44) (Supp. Table 5). Given similar performance and greater parsimony, we selected the CCA model for final score development.

## Discussion

This study introduces a simple risk score for predicting hospital admission, designed to enhance triage decision-making and improve outcomes for older adults in the emergency department (ED). Unlike existing models, which often focus solely on predicting admissions, our approach identifies patients at high risk for admission as well as those at low risk for discharge, helping to mitigate the negative impact of prolonged ED stays on this vulnerable population. By leveraging routinely available triage data, the model supports timely decision-making, which is critical for older adults who are particularly sensitive to delays in care.

Predicting hospital admissions for older adults presents distinct challenges. Chronological age alone is a limited predictor of health status due to heterogeneous nature of aging, and while vital signs are informative, tools like the Modified Early Warning Score (MEWS) often fall short in identifying severely ill older patients with atypical presentations because of altered physiology, polypharmacy, and comorbidities [26–28]. Social and cognitive factors further complicate admission decisions, as frail or cognitively impaired patients often cannot advocate for themselves “silent by proxy” [29]. Additionally, crucial lab results and imaging findings are usually unavailable during the initial assessment. Recognizing these limitations, we developed a triage-level model using demographics, presenting complaints, vital signs, and comorbidities, enabling risk assessment early in the ED visit.

Our model predicts hospital admission at the triage level for older adult patients, relying solely on initial assessment data to enable early forecasting of patient disposition. By excluding post-encounter data such as lab results or imaging, it focuses on early risk stratification for resource allocation but at the cost of reduced predictive accuracy. This limitation is magnified by the high baseline admission rates observed in older adults, which decrease risk variability and make discrimination more challenging when adapting models originally designed for the general population [30]. 

To address these challenges, our model adopts a threshold-based strategy, emphasizing sensitivity for low-risk discharges (91% accuracy) and specificity for high-risk admissions (58% accuracy). This tailored approach enhances clinical utility despite moderate overall accuracy. Our model dichotomizes vital signs using established clinical criteria, including systemic inflammatory response syndrome (SIRS) parameters (heart rate > 90 beats/min, respiratory rate > 20 breaths/min, temperature < 36 °C or > 38 °C), and other validated thresholds from emergency medicine literature [31]. These objective cutoffs align with widely-used risk assessment tools such as the Senior Triage tool (S-TRIAGE), the quick Sequential (Sepsis-related) Organ Failure Assessment (qSOFA), and the Manchester Triage System, which have demonstrated predictive value for adverse outcomes [32–34]. While these metrics have demonstrated predictive value across various clinical settings, older adult patients often present with complexities not fully captured by vital signs alone. To address this gap, the model incorporates additional parameters such as chief complaints, comorbidities, and mode of arrival, enabling a more comprehensive risk stratification at ED presentation. The digital implementation of the model supports rapid, evidence-based guidance, making it a valuable adjunct to existing triage workflows. Additionally, Its simplicity makes it particularly suitable for widespread adoption in resource-limited settings where it can complement commonly used tools like the Interagency Integrated Triage Tool (IITT) [35]. 

It is important to acknowledge that the model’s predictions reflect observed triage decisions rather than direct assessments of care quality. Further studies should evaluate its impact on clinical decision-making, patient outcomes, and ED operations, including potential contributions to more efficient resource allocation. The model is available as an interactive tool online: https://admission.shinyapps.io/AdmissionRiscScore/.

Despite its strengths, this study has several important limitations. First, the retrospective analysis of NHAMC data did not capture critical older adult-specific variables such as functional status (e.g., activities of daily living, mobility), cognitive function, and social support networks which can influence admission decisions. Second, the cross-sectional nature of the data prevented us from analyzing dynamic changes in patient condition during ED stays, which could affect disposition decisions. Third, our primary outcome of hospital admission was subject to variability in physician decision-making and institutional factors such as bed availability and resource constraints. Fourth, by using pre-COVID-19 data to ensure consistency in healthcare delivery patterns, our findings may not fully generalize to current emergency care practices which have evolved in response to the pandemic. Fifth, while our model demonstrated robust internal validation, it requires external validation across diverse healthcare settings and patient populations to confirm its clinical utility. Finally, our inclusion criterion of chronological age ≥ 60 years may oversimplify the complex relationship between aging and healthcare needs, as it does not account for variations in biological aging rates and frailty status that can significantly impact ED disposition decisions [36, 37]. 

In conclusion, this study offers a practical risk prediction tool that supports early, data-driven triage decisions for older adults. Future research should focus on prospective validation across diverse settings, assessment of long-term calibration, and integration within existing triage frameworks to optimize care for older adults prioritization in Emergency Departments.

## Electronic supplementary material

Below is the link to the electronic supplementary material.

Supplementary Material 1

## Data Availability

No datasets were generated or analysed during the current study.
