# Supplementary material for: Development of an emergency department triage tool to predict admission or discharge for older adults
Source: Int J Emerg Med. 2025 Feb 14;18:26. doi: 10.1186/s12245-025-00825-3 (PMC11827304; doi:10.1186/s12245-025-00825-3)
Supplement: Supplementary file 1 — Supplementary Material 1 [file 12245_2025_825_MOESM1_ESM.docx]

**Supp. Table 1: TRIPOD Checklist: Prediction Model Development**

| **Section/Topic. Item Checklist Item Page** | | | |
| --- | --- | --- | --- |
| **Title and abstract** | | | |
| Title | 1 | Identify the study as developing and/or validating a multivariable prediction model, the target population, and the outcome to be predicted. | 1 |
| Abstract | 2 | Provide a summary of objectives, study design, setting, participants, sample size, predictors, outcome, statistical analysis, results, and conclusions. | 2 |
| **Introduction** | | | |
| Background and objectives | 3a | Explain the medical context (including whether diagnostic or prognostic) and rationale for developing or validating the multivariable prediction model, including references to existing models. | 3 |
|  | 3b | Specify the objectives, including whether the study describes the development or validation of the model or both. | 3 |
| **Methods** | | | |
| Source of data | 4a | Describe the study design or source of data (e.g., randomized trial, cohort, or registry data), separately for the development and validation data sets, if applicable. | 3 |
|  | 4b | Specify the key study dates, including start of accrual; end of accrual; and, if applicable, end of follow-up. | 3 |
| Participants | 5a | Specify key elements of the study setting (e.g., primary care, secondary care, general population) including number and location of centres. | 4 |
|  | 5b | Describe eligibility criteria for participants. | 4 |
|  | 5c | Give details of treatments received, if relevant. | NA |
| Outcome | 6a | Clearly define the outcome that is predicted by the prediction model, including how and when assessed. | 4 |
|  | 6b | Report any actions to blind assessment of the outcome to be predicted. | NA |
| Predictors | 7a | Clearly define all predictors used in developing or validating the multivariable  prediction model, including how and when they were measured. | 4 |
|  | 7b | Report any actions to blind assessment of predictors for the outcome and other predictors. | NA |
| Sample size | 8 | Explain how the study size was arrived at. | S.6 |
| Missing data | 9 | Describe how missing data were handled (e.g., complete-case analysis, single imputation, multiple imputation) with details of any imputation method. | 4 |
| Statistical analysis methods | 10a | Describe how predictors were handled in the analyses. | 4 |
|  | 10b | Specify type of model, all model-building procedures (including any predictor selection), and method for internal validation. | 4,5 |
|  | 10d | Specify all measures used to assess model performance and, if relevant, to compare multiple models. | 5 |
| Risk groups | 11 | Provide details on how risk groups were created, if done. | 5 |
| **Results** | | | |
| Participants | 13a | Describe the flow of participants through the study, including the number of participants with and without the outcome and, if applicable, a summary of the follow-up time. A diagram may be helpful. | 5 |
|  | 13b | Describe the characteristics of the participants (basic demographics, clinical features, available predictors), including the number of participants with missing data for predictors and outcome. | 5 |
| Model development | 14a | Specify the number of participants and outcome events in each analysis. | 5 |
|  | 14b | If done, report the unadjusted association between each candidate predictor and outcome. | 9 |
| Model specification | 15a | Present the full prediction model to allow predictions for individuals (i.e., all regression coefficients, and model intercept or baseline survival at a given time point). | Fig3 |
|  | 15b | Explain how to the use the prediction model. | 7 & Fig3 |
| Model performance | 16 | Report performance measures (with CIs) for the prediction model. | 6 |
| **Discussion** | | | |
| Limitations | 18 | Discuss any limitations of the study (such as nonrepresentative sample, few events per predictor, missing data). | 7 |
| Interpretation | 19b | Give an overall interpretation of the results, considering objectives, limitations, and results from similar studies, and other relevant evidence. | 7 |
| Implications | 20 | Discuss the potential clinical use of the model and implications for future research. | 7 |
| **Other information** | | | |
| Supplementary information | 21 | Provide information about the availability of supplementary resources, such as study protocol, Web calculator, and data sets. | 7 |
| Funding | 22 | Give the source of funding and the role of the funders for the present study. | 8 |

**Supp. Table 2: Study covariates in train and test data.**

| Label | Overall | Test Cohort | Training Cohort | SMD |
| --- | --- | --- | --- | --- |
| n | 13431 | 4030 | 9401 |  |
| Male Sex | 5884 (43.8) | 1764 (43.8) | 4120 (43.8) | <0.01 |
| Age in years | 72.0 [65.0, 81.0] | 71.0 [65.0, 80.0] | 72.0 [65.0, 81.0] | 0.02 |
| **Race and Ethnicity** |  |  |  | 0.05 |
| Non-Hispanic White | 9607 (71.5) | 2933 (72.8) | 6674 (71.0) |  |
| Non-Hispanic Black | 2136 (15.9) | 596 (14.8) | 1540 (16.4) |  |
| Hispanic | 1110 (8.3) | 342 (8.5) | 768 (8.2) |  |
| Non-Hispanic Other | 578 (4.3) | 159 (3.9) | 419 (4.5) |  |
| Nursing home resident | 720 (5.4) | 217 (5.4) | 503 (5.4) | <0.01 |
| Initial ED visit | 804 (6.0) | 247 (6.1) | 557 (5.9) | 0.01 |
| Arrived by Ambulance | 3943 (29.4) | 1145 (28.4) | 2798 (29.8) | 0.03 |
| Weekend Admission | 3550 (26.4) | 1084 (26.9) | 2466 (26.2) | 0.02 |
| **Time of ED visit** |  |  |  | 0.05 |
| 7:00 AM-7:00 PM | 10293 (76.6) | 3103 (77.0) | 7190 (76.5) |  |
| 8:00 PM- 1:00 AM | 2186 (16.3) | 677 (16.8) | 1509 (16.1) |  |
| 2:00 AM-6:00 AM | 952 (7.1) | 250 (6.2) | 702 (7.5) |  |
| **Season** |  |  |  | 0.02 |
| Winter | 3355 (25.0) | 1002 (24.9) | 2353 (25.0) |  |
| Spring | 3629 (27.0) | 1111 (27.6) | 2518 (26.8) |  |
| Summer | 3125 (23.3) | 923 (22.9) | 2202 (23.4) |  |
| Autumn | 3322 (24.7) | 994 (24.7) | 2328 (24.8) |  |
| **Insurance Type** |  |  |  |  |
| Medicare insurance | 9094 (67.7) | 2688 (66.7) | 6406 (68.1) | 0.03 |
| Private insurance | 5318 (39.6) | 1626 (40.3) | 3692 (39.3) | 0.02 |
| Medicaid insurance | 2453 (18.3) | 737 (18.3) | 1716 (18.3) | <0.01 |
| Residency program in ED | 3644 (27.1) | 1089 (27.0) | 2555 (27.2) | <0.01 |
| ED bed Coordinator present | 10188 (75.9) | 3032 (75.2) | 7156 (76.1) | 0.02 |
| Admitted pts every boarded > 2 hrs. | 10432 (77.7) | 3114 (77.3) | 7318 (77.8) | 0.01 |
| Wait time to first provider in ED (Minutes) | 18.0 [6.0, 35.2] | 18.0 [7.0, 35.2] | 18.0 [6.0, 35.2] | 0.01 |
| Wait time before first provider ≥ 1 hour | 1780 (13.3) | 539 (13.4) | 1241 (13.2) | 0.01 |
| **Chronic Conditions** |  |  |  |  |
| History of Pulmonary Embolism | 517 (3.8) | 167 (4.1) | 350 (3.7) | 0.02 |
| History of Heart disease | 3659 (27.2) | 1080 (26.8) | 2579 (27.4) | 0.01 |
| Alzheimer's disease/Dementia | 788 (5.9) | 248 (6.2) | 540 (5.7) | 0.02 |
| Asthma | 1116 (8.3) | 321 (8.0) | 795 (8.5) | 0.02 |
| Cancer | 1612 (12.0) | 469 (11.6) | 1143 (12.2) | 0.02 |
| History of Stroke or TIA | 1371 (10.2) | 415 (10.3) | 956 (10.2) | <0.01 |
| Chronic Kidney Disease | 1246 (9.3) | 380 (9.4) | 866 (9.2) | 0.01 |
| Chronic Obstructive Pulmonary Disease | 2114 (15.7) | 605 (15.0) | 1509 (16.1) | 0.03 |
| Depression | 1785 (13.3) | 515 (12.8) | 1270 (13.5) | 0.02 |
| End stage renal disease | 288 (2.1) | 93 (2.3) | 195 (2.1) | 0.02 |
| Obesity BMI > 30 | 944 (7.0) | 299 (7.4) | 645 (6.9) | 0.02 |
| Obstructive Sleep Apnea | 750 (5.6) | 212 (5.3) | 538 (5.7) | 0.02 |
| Substance abuse or dependence | 517 (3.8) | 145 (3.6) | 372 (4.0) | 0.02 |
| Alcohol misuse, abuse, or dependence | 418 (3.1) | 128 (3.2) | 290 (3.1) | 0.01 |
| Diabetes Mellitus | 3778 (28.1) | 1067 (26.5) | 2711 (28.8) | 0.05 |
| Number of chronic conditions | 2.0 [1.0, 4.0] | 2.0 [1.0, 4.0] | 2.0 [1.0, 4.0] | 0.02 |
| **Presenting condition** |  |  |  |  |
| Fracture or dislocation | 36 (0.3) | 5 (0.1) | 31 (0.3) | 0.04 |
| Motor Vehicle Accident | 66 (0.5) | 22 (0.5) | 44 (0.5) | 0.01 |
| Accident including falls | 440 (3.3) | 138 (3.4) | 302 (3.2) | 0.01 |
| Non-specific body pain/cramp/stiffness | 100 (0.7) | 22 (0.5) | 78 (0.8) | 0.03 |
| Back pain | 1329 (9.9) | 393 (9.8) | 936 (10.0) | 0.01 |
| Tiredness, exhaustion and General weakness | 478 (3.6) | 137 (3.4) | 341 (3.6) | 0.01 |
| Fainting not LOC | 160 (1.2) | 57 (1.4) | 103 (1.1) | 0.03 |
| Chest pain, pressure or discomfort | 895 (6.7) | 292 (7.2) | 603 (6.4) | 0.03 |
| Shortness of breath | 964 (7.2) | 276 (6.8) | 688 (7.3) | 0.02 |
| Edema including leg swelling | 86 (0.6) | 26 (0.6) | 60 (0.6) | <0.01 |
| Cough | 344 (2.6) | 127 (3.2) | 217 (2.3) | 0.05 |
| Throat pain irritation and swelling | 77 (0.6) | 28 (0.7) | 49 (0.5) | 0.02 |
| Neurological symptoms | 218 (1.6) | 55 (1.4) | 163 (1.7) | 0.03 |
| Headache | 269 (2.0) | 78 (1.9) | 191 (2.0) | 0.01 |
| Vertigo | 408 (3.0) | 130 (3.2) | 278 (3.0) | 0.02 |
| Abnormal sensation | 74 (0.6) | 15 (0.4) | 59 (0.6) | 0.04 |
| Skin rash | 83 (0.6) | 22 (0.5) | 61 (0.6) | 0.01 |
| Dysuria, hematuria or urinary retention | 221 (1.6) | 72 (1.8) | 149 (1.6) | 0.02 |
| Abdominal pain | 845 (6.3) | 259 (6.4) | 586 (6.2) | 0.01 |
| GI bleeding | 66 (0.5) | 17 (0.4) | 49 (0.5) | 0.01 |
| Nausea or vomiting | 324 (2.4) | 101 (2.5) | 223 (2.4) | 0.01 |
| Constipation | 90 (0.7) | 22 (0.5) | 68 (0.7) | 0.02 |
| Diarrhea | 116 (0.9) | 36 (0.9) | 80 (0.9) | <0.01 |
| Behavioral disturbance | 39 (0.3) | 13 (0.3) | 26 (0.3) | 0.01 |
| Psychiatric problem | 94 (0.7) | 28 (0.7) | 66 (0.7) | <0.01 |
| **Abnormal Vitals** |  |  |  |  |
| Temp < 96.8 or > 100.4°F | 619 (4.6) | 195 (4.8) | 424 (4.5) | 0.02 |
| SBP < 100 or ≥ 180 or DBP < 60 or ≥110 | 3357 (25.0) | 1035 (25.7) | 2322 (24.7) | 0.02 |
| HR < 60 or > 90 | 4527 (33.7) | 1368 (33.9) | 3159 (33.6) | 0.01 |
| Respiratory rate < 11 or > 20 | 1406 (10.5) | 426 (10.6) | 980 (10.4) | <0.01 |
| Hypoxia (O2 Sat < 90) | 361 (2.7) | 111 (2.8) | 250 (2.7) | 0.01 |
| Pain scale > 7 | 2447 (18.2) | 719 (17.8) | 1728 (18.4) | 0.01 |

**Supp. Table 3: Predicted Hospital Admission Risk by Risk Score**

| Risk Score | Predicted Admission Risk | Lower CI | Upper CI |
| --- | --- | --- | --- |
| 0 | 8.6% | 7.8% | 9.3% |
| 1 | 11.2% | 10.4% | 12% |
| 2 | 14.4% | 13.6% | 15.2% |
| 3 | 18.4% | 17.6% | 19.3% |
| 4 | 23.2% | 22.3% | 24.2% |
| 5 | 28.9% | 27.8% | 30% |
| 6 | 35.3% | 33.9% | 36.6% |
| 7 | 42.2% | 40.5% | 43.9% |
| 8 | 49.5% | 47.5% | 51.5% |
| 9 | 56.8% | 54.4% | 59.1% |
| 10 | 63.8% | 61.2% | 66.3% |
| 11 | 70.2% | 67.6% | 72.9% |
| 12 | 76% | 73.4% | 78.6% |
| 13 | 80.9% | 78.5% | 83.4% |
| 14 | 85.1% | 82.8% | 87.3% |
| 15 | 88.4% | 86.4% | 90.4% |
| 16 | 91.1% | 89.4% | 92.8% |
| 17 | 93.2% | 91.8% | 94.6% |
| 18 | 94.8% | 93.7% | 96% |
| 19 | 96.1% | 95.1% | 97.1% |
| 20 | 97.1% | 96.3% | 97.9% |
| 21 | 97.8% | 97.2% | 98.4% |
| 22 | 98.3% | 97.8% | 98.9% |
| 23 | 98.8% | 98.4% | 99.2% |
| 24 | 99.1% | 98.8% | 99.4% |
| 25 | 99.3% | 99.1% | 99.6% |
| 26 | 99.5% | 99.3% | 99.7% |
| 27 | 99.6% | 99.5% | 99.8% |
| 28 | 99.7% | 99.6% | 99.8% |
| 29 | 99.8% | 99.7% | 99.9% |
| 30 | 99.8% | 99.8% | 99.9% |
| 31 | 99.9% | 99.8% | 99.9% |
| 32 | 99.9% | 99.9% | 100% |
| 33 | 99.9% | 99.9% | 100% |

**Supp. Table 4: Calibration Plot of Predicted vs. Observed Admission Probabilities by Decile (From Test dataset)**

| Decile | Predicted | Observed |
| --- | --- | --- |
| 10% | 8.9 | 9.2 |
| 20% | 9.2 | 8.9 |
| 30% | 12.4 | 10.9 |
| 40% | 13.8 | 12.4 |
| 50% | 17.4 | 13.4 |
| 60% | 20.0 | 22.3 |
| 70% | 24.9 | 26.1 |
| 80% | 30.2 | 32.3 |
| 90% | 39.6 | 45.9 |
| 100% | 60.4 | 55.6 |

**Supp. Table 5: Comparison of Complete Case Analysis (Model 1, n=13,431) versus Multiple Imputation Analysis (Model 2, n=16,028) for ED Admission Risk Score Development.**

|  | **Model 1** | | **Model 2** | |
| --- | --- | --- | --- | --- |
| Variable | OR (95% CI) p-value | Weight | OR (95% CI) p-value | Weight |
| GI bleeding | 5.33 (2.95 - 9.68) <0.001 | 5.7 | 4.6 (2.51 - 8.47) <0.001 | 8.7 |
| Neurological symptoms | 2.66 (1.89 - 3.75) <0.001 | 3.3 | 2.17 (1.6 - 2.92) <0.001 | 4.4 |
| Arrived by Ambulance | 2.44 (2.2 - 2.72) <0.001 | 3.0 | 2.55 (2.32 - 2.81) <0.001 | 5.4 |
| Shortness of breath | 2.28 (1.9 - 2.74) <0.001 | 2.8 | 2.55 (2.16 - 3.01) <0.001 | 5.4 |
| Temp < 96.8 or > 100.4°F | 2.15 (1.73 - 2.68) <0.001 | 2.6 | 2.33 (1.9 - 2.86) <0.001 | 4.9 |
| Chronic Kidney Disease | 2.11 (1.8 - 2.47) <0.001 | 2.5 | 1.96 (1.69 - 2.27) <0.001 | 3.9 |
| Chest pain, pressure or discomfort | 1.97 (1.62 - 2.38) <0.001 | 2.3 | 1.79 (1.51 - 2.13) <0.001 | 3.3 |
| Respiratory rate < 11 or > 20 | 1.77 (1.51 - 2.07) <0.001 | 1.9 | 1.56 (1.35 - 1.81) <0.001 | 2.6 |
| Cancer | 1.72 (1.49 - 1.98) <0.001 | 1.8 | 1.63 (1.42 - 1.86) <0.001 | 2.8 |
| History of Stroke or TIA | 1.67 (1.43 - 1.95) <0.001 | 1.8 | 1.38 (1.19 - 1.59) <0.001 | 1.8 |
| Hypoxia (O_2_sat < 90) | 1.65 (1.24 - 2.2) <0.001 | 1.7 | 2.14 (1.68 - 2.73) <0.001 | 4.4 |
| HR < 60 or > 90 | 1.61 (1.44 - 1.79) <0.001 | 1.6 | 1.57 (1.42 - 1.73) <0.001 | 2.6 |
| CAD or CHF | 1.5 (1.34 - 1.67) <0.001 | 1.4 | 1.52 (1.37 - 1.69) <0.001 | 2.4 |
| SBP < 100 or ≥ 180 or DBP < 60 or ≥110 | 1.34 (1.2 - 1.5) <0.001 | 1.0 | 1.27 (1.14 - 1.41) <0.001 | 1.4 |
| Obesity BMI > 30 |  |  | 1.57 (1.32 - 1.87) <0.001 | 2.6 |
| Age ≥ 75 |  |  | 1.19 (1.08 - 1.31) <0.001 | 1.0 |
| (Intercept) | 0.09 (0.08 - 0.1) <0.001 |  | 0.09 (0.08 - 0.1) <0.001 |  |

Both models showed similar discrimination (AUC 0.73) despite differences in variable selection and weights. Variables are ordered by descending odds ratios in Model 1.

**Supp. Figure 1a: Study Population and Sample Selection**

**
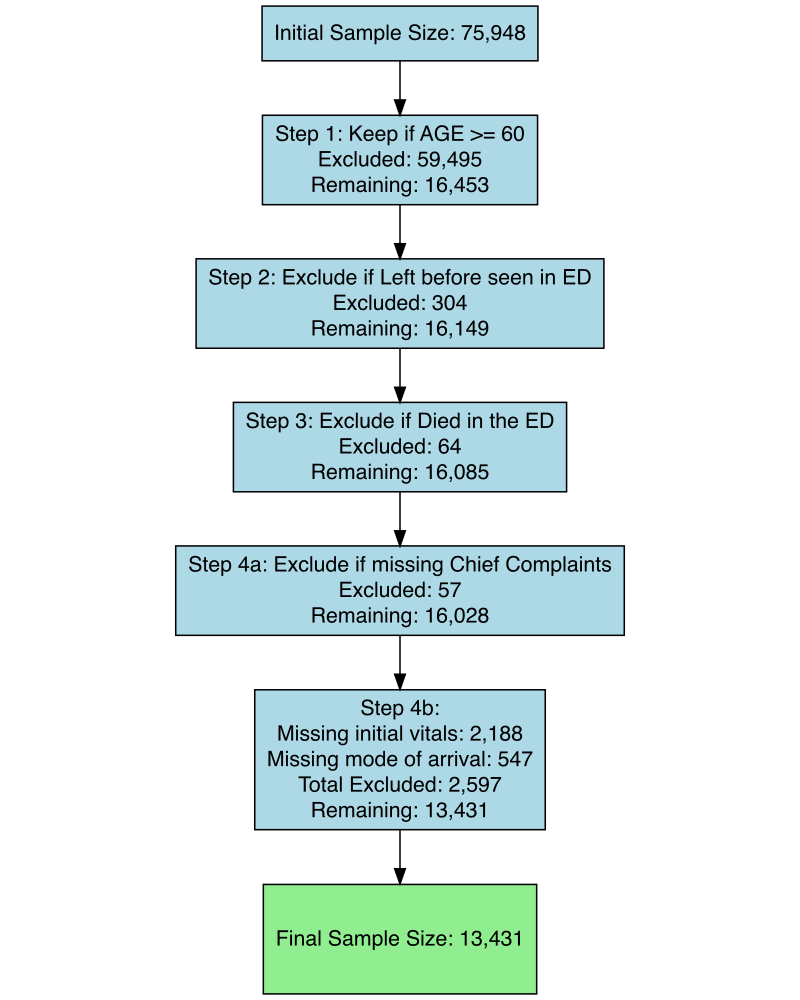
**

**Supp. Figure 1b:**

**
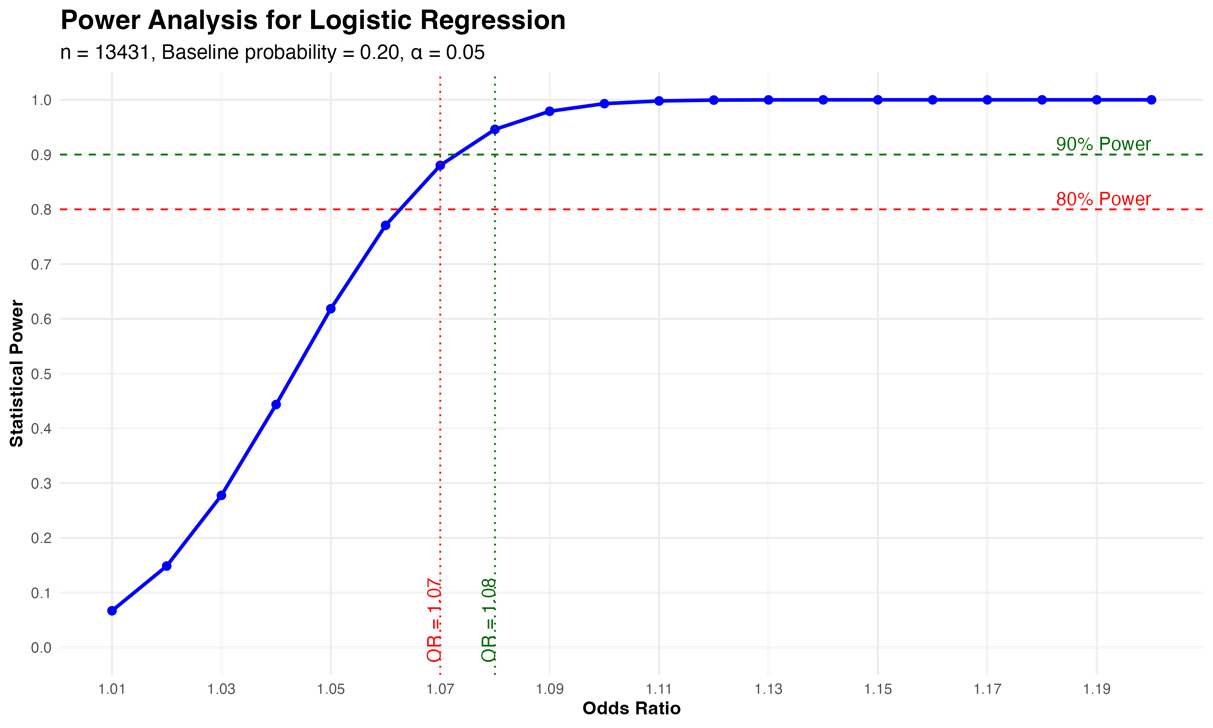
**

**Supp. Figure 2: Missing Data distribution.**

**
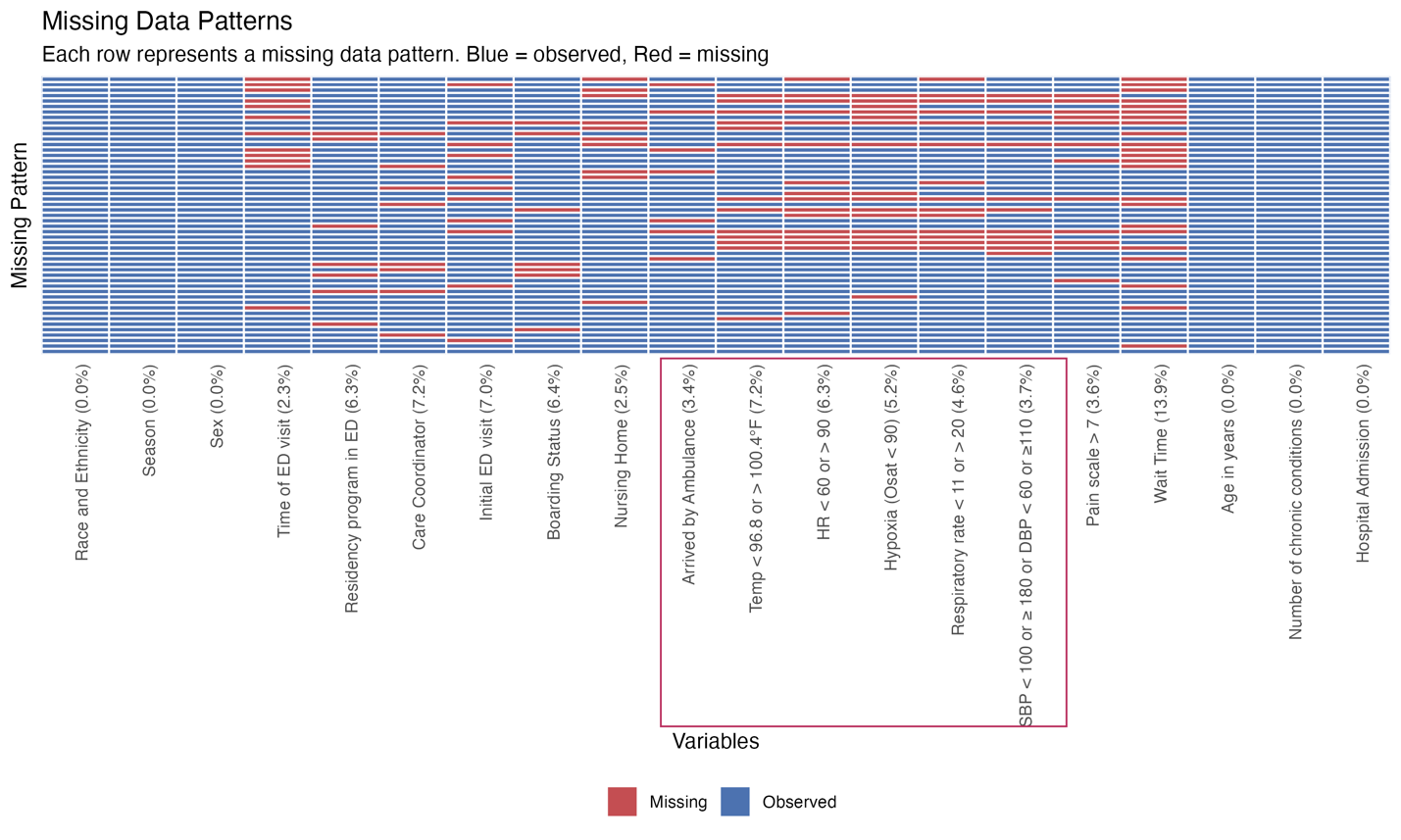
**

Missing Data Patterns in ED Admission Variables. Each row represents a distinct missing data pattern, with blue indicating observed values and red indicating missing values. The boxed variables (vital signs and mode of transportation) had 3-7% missing values and were addressed using multiple imputation in sensitivity analyses.

**Supp Figure 3: LASSO Coefficient path**

**
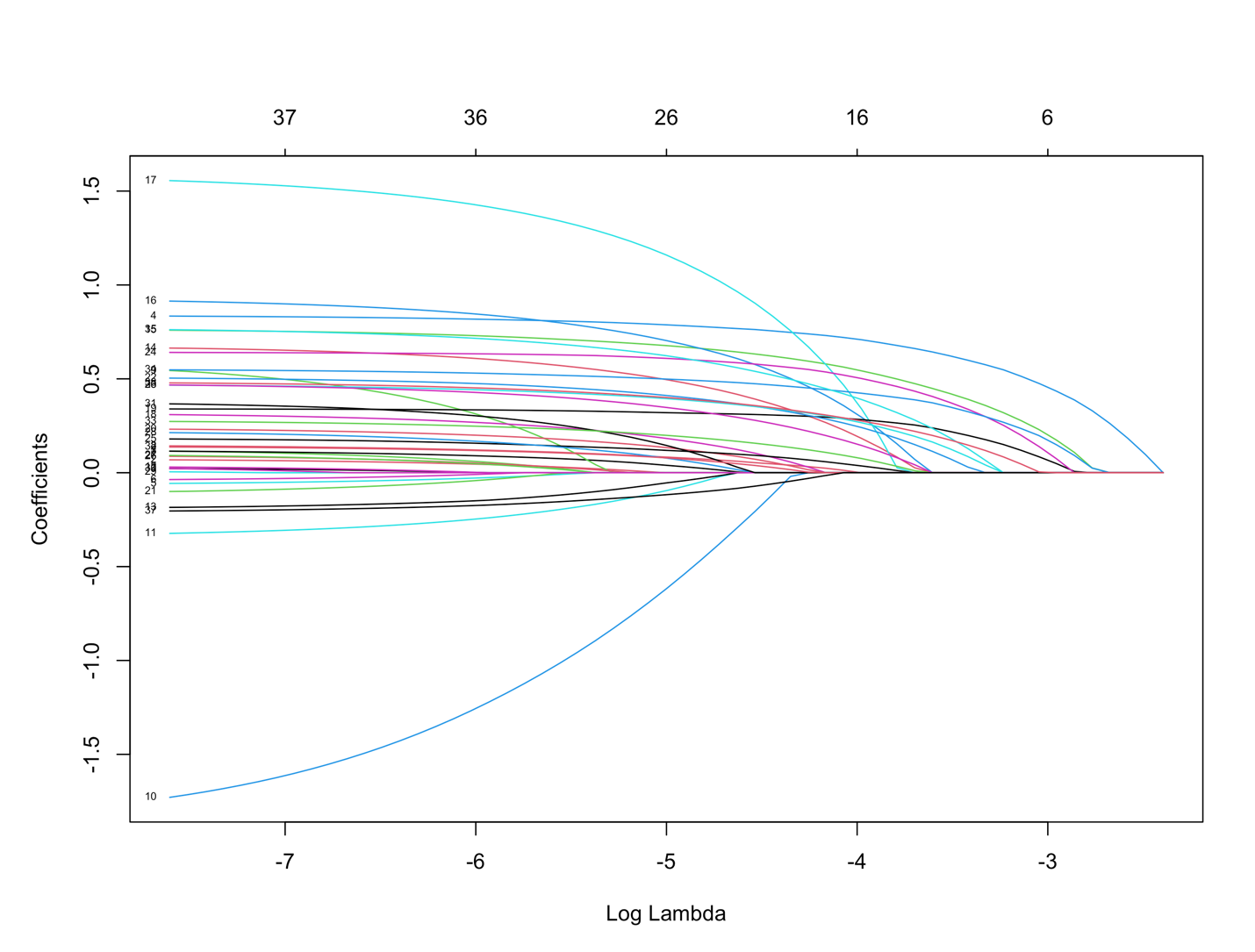
**

The LASSO (Least Absolute Shrinkage and Selection Operator) coefficient path plot visually represents how coefficients change as the regularization parameter 𝜆 varies. On the x-axis, the log of 𝜆 ranges from high regularization (left) to low (right). The y-axis shows the values of the coefficients for each predictor, with each colored line representing a different variable. When λ is large, all coefficients are near zero, excluding variables from the model. As λ decreases, coefficients increase from zero, indicating the inclusion of more variables. At very small λ values, the model approximates ordinary least squares estimates, including many predictors. This plot highlights the LASSO method's ability to perform variable selection and regularization. Non-zero coefficients at specific λ values identify important variables. The plot aids in understanding the trade-off between model complexity and λ, guiding the selection of a λ value that balances simplicity and accuracy, typically determined through cross-validation.

**Supp Figure 4: Cross-Validation Curve for LASSO**


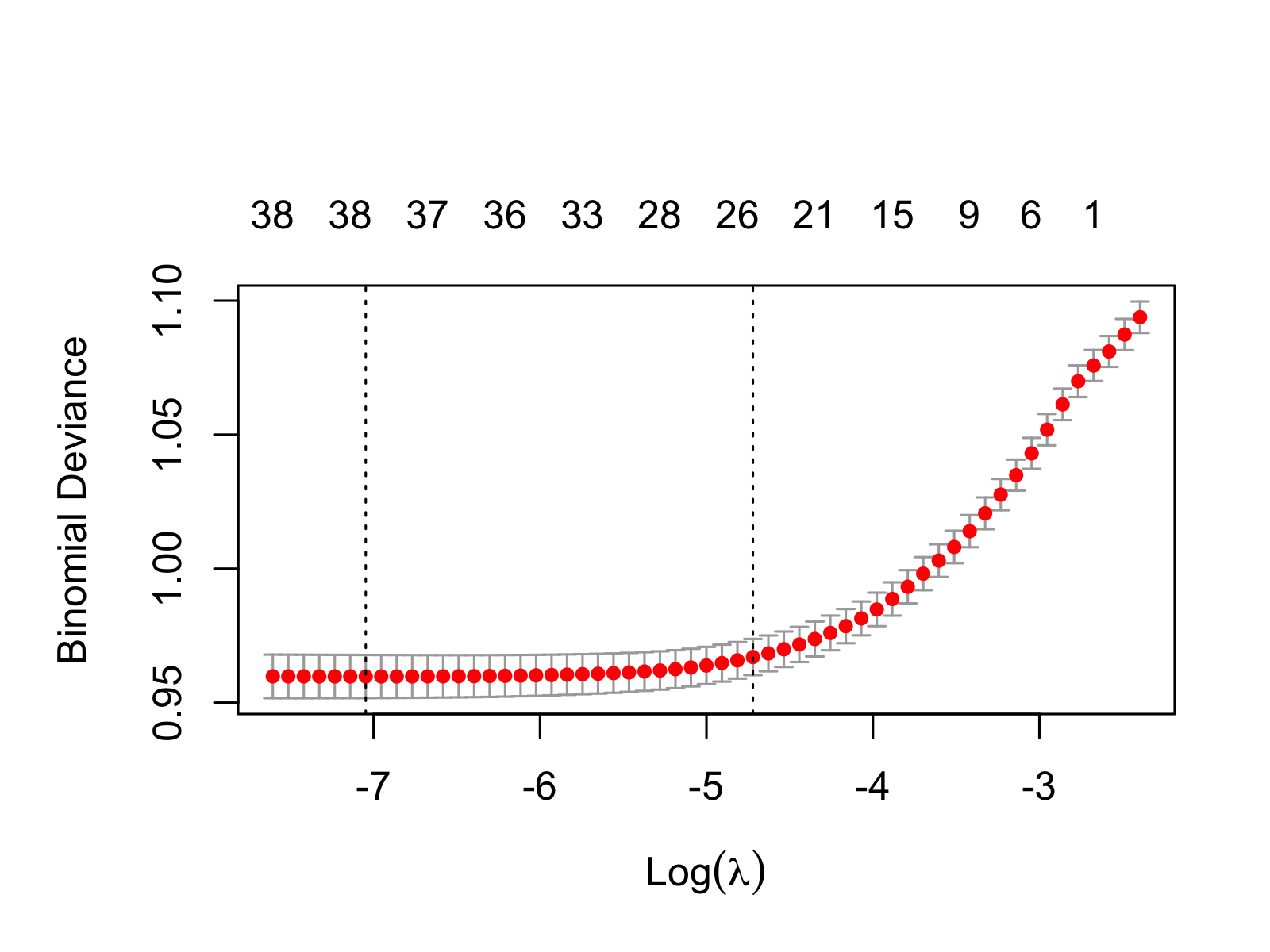


The Cross-Validation Curve for LASSO plot illustrates the relationship between the log of the regularization parameter 𝜆 and the binomial deviance, a measure of model performance. The x-axis represents the log of λ, ranging from high regularization (left) to low regularization (right). The y-axis shows the binomial deviance, where lower values indicate better model fit. The red dots represent the binomial deviance values obtained from cross-validation for each λ, with error bars indicating the variability. As 𝜆 decreases, the deviance initially remains low and stable, suggesting a good model fit with effective regularization. However, as λ continues to decrease further (moving to the right), the deviance increases, indicating overfitting due to reduced regularization. The vertical dashed lines typically mark the optimal λ values. The first line (left) often corresponds to the 𝜆 that minimizes the cross-validated deviance, while the second line (right) represents the largest λ within one standard error of the minimum deviance. These points help select a λ that balances model complexity and predictive accuracy, ensuring the model is neither overfitted nor underfitted. Overall, this plot aids in identifying the optimal 𝜆 for the LASSO model by balancing bias and variance, guiding the selection process for the best-performing and most generalizable model.

**Supp. Figure 5: Variable selection by LASSO**

**
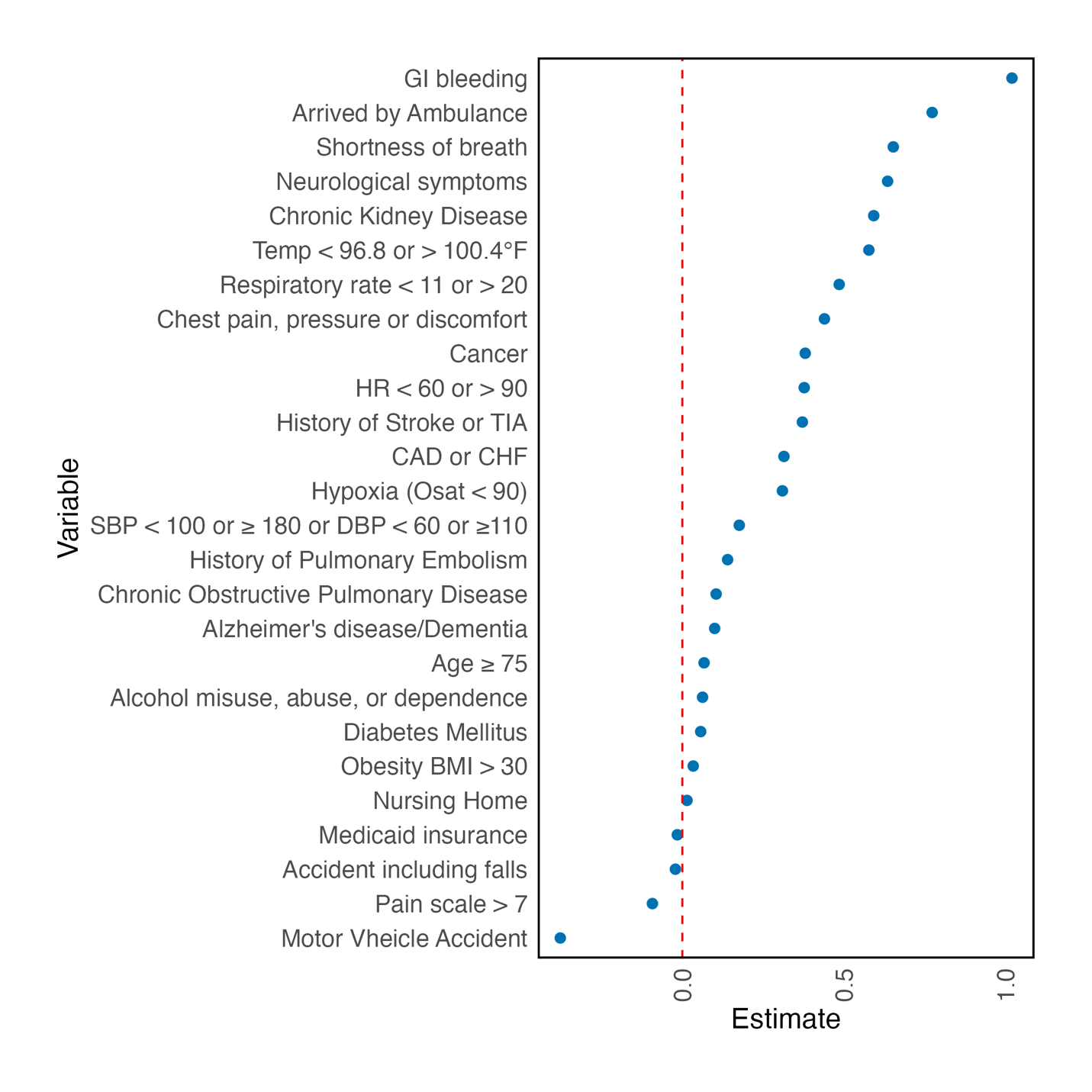
**

The plot shows key predictors and their relative importance from a LASSO regression model. Each point represents a variable’s effect size, with the y-axis listing variables and the x-axis showing coefficient magnitude. Positive coefficients indicate a direct relationship, while negative coefficients indicate an inverse relationship. The red dashed line at zero highlights significant non-zero effects.

**Supp. Figure 6: LASSO Feature Importance Plot**


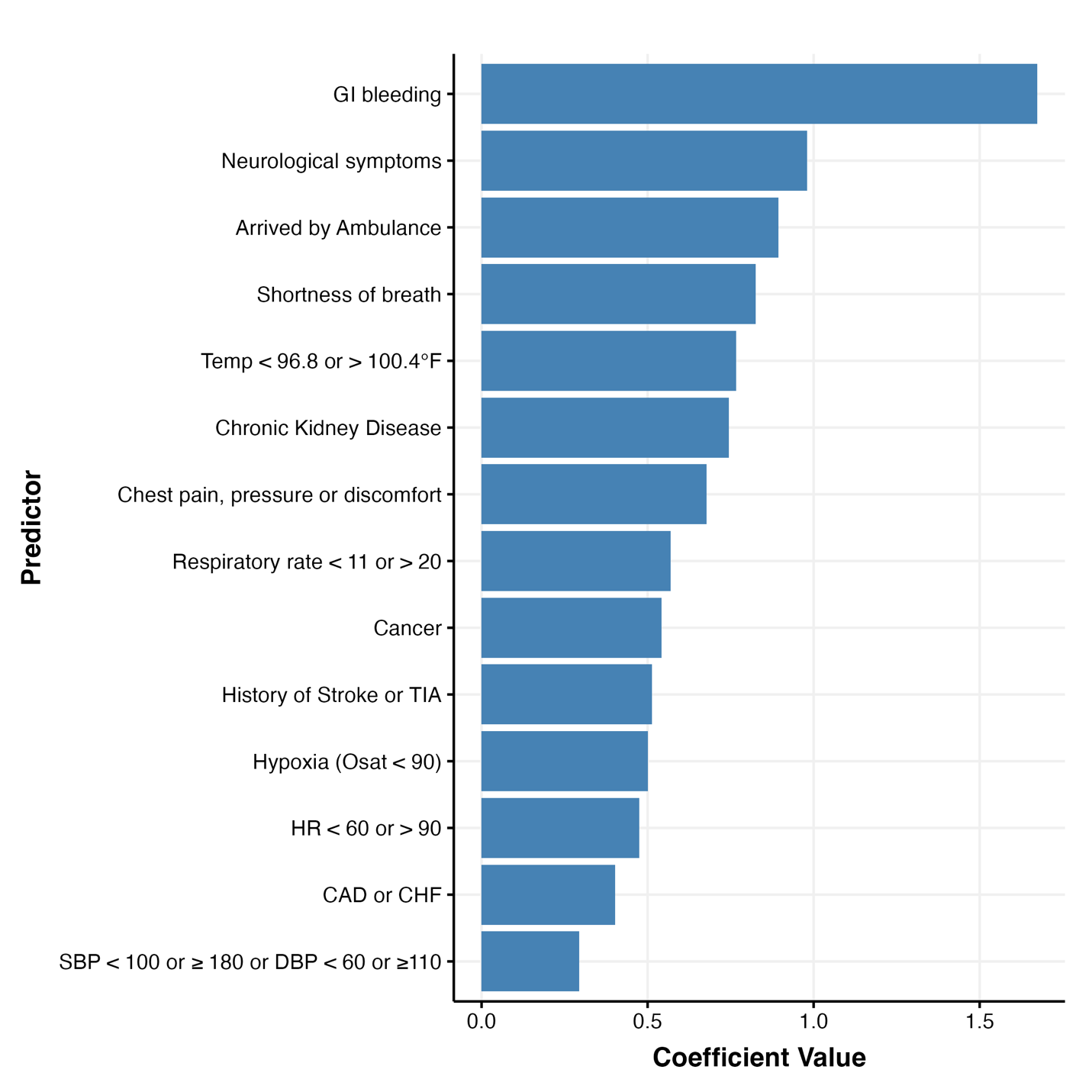


Feature importance plot highlights the most significant factors in the model, showing the impact of various predictors based on their coefficient values from a LASSO regression model. Each bar represents a predictor, with length indicating effect magnitude.

**Supp Figure 7: Density plot of risk for admission score in those admitted vs not admitted within testing and training datasets during the study period.**


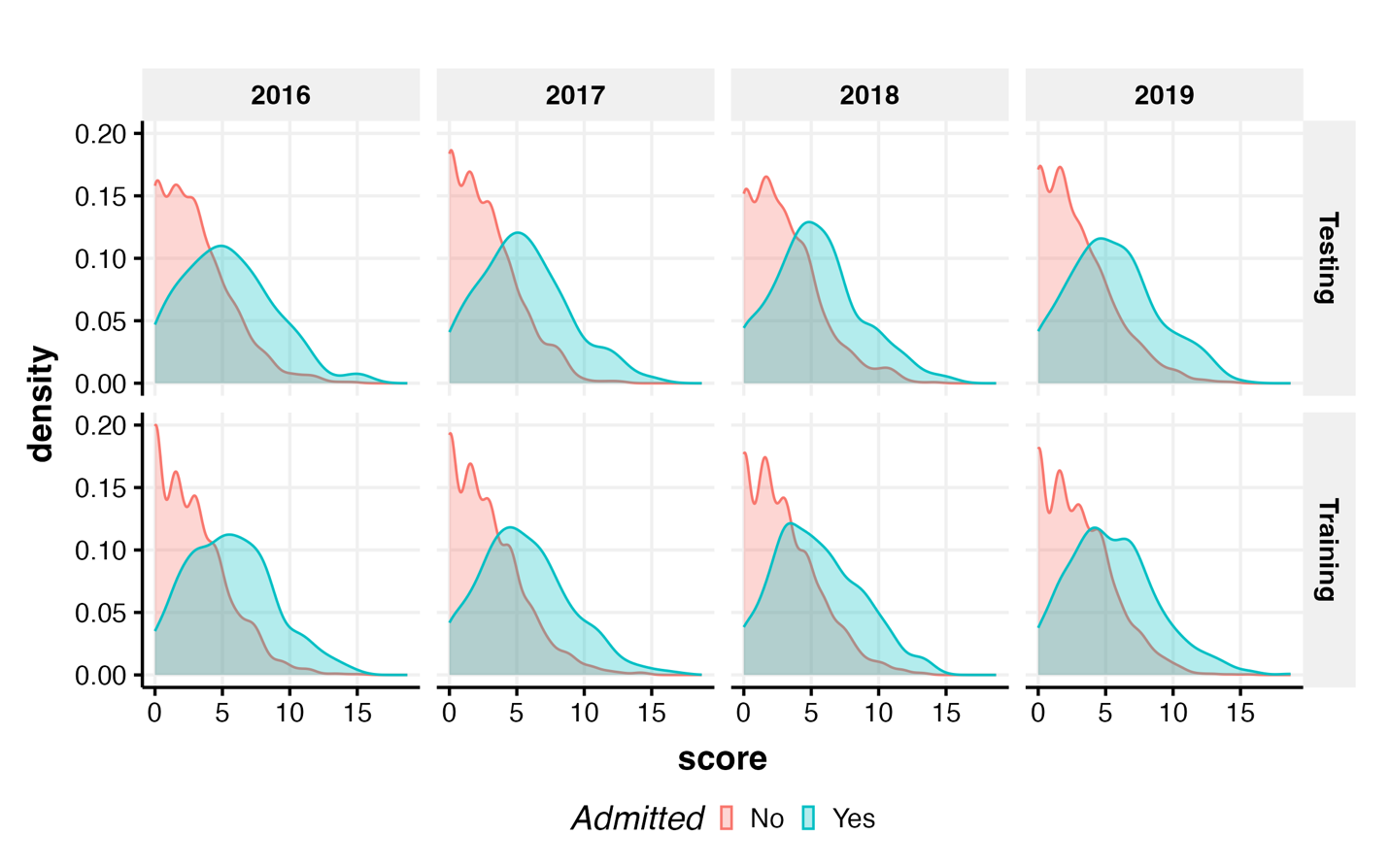


**Supp. Figure 8: Point-Based Nomogram for Estimating Hospital Admission Probability Among Older Adults in the Emergency Department**

**
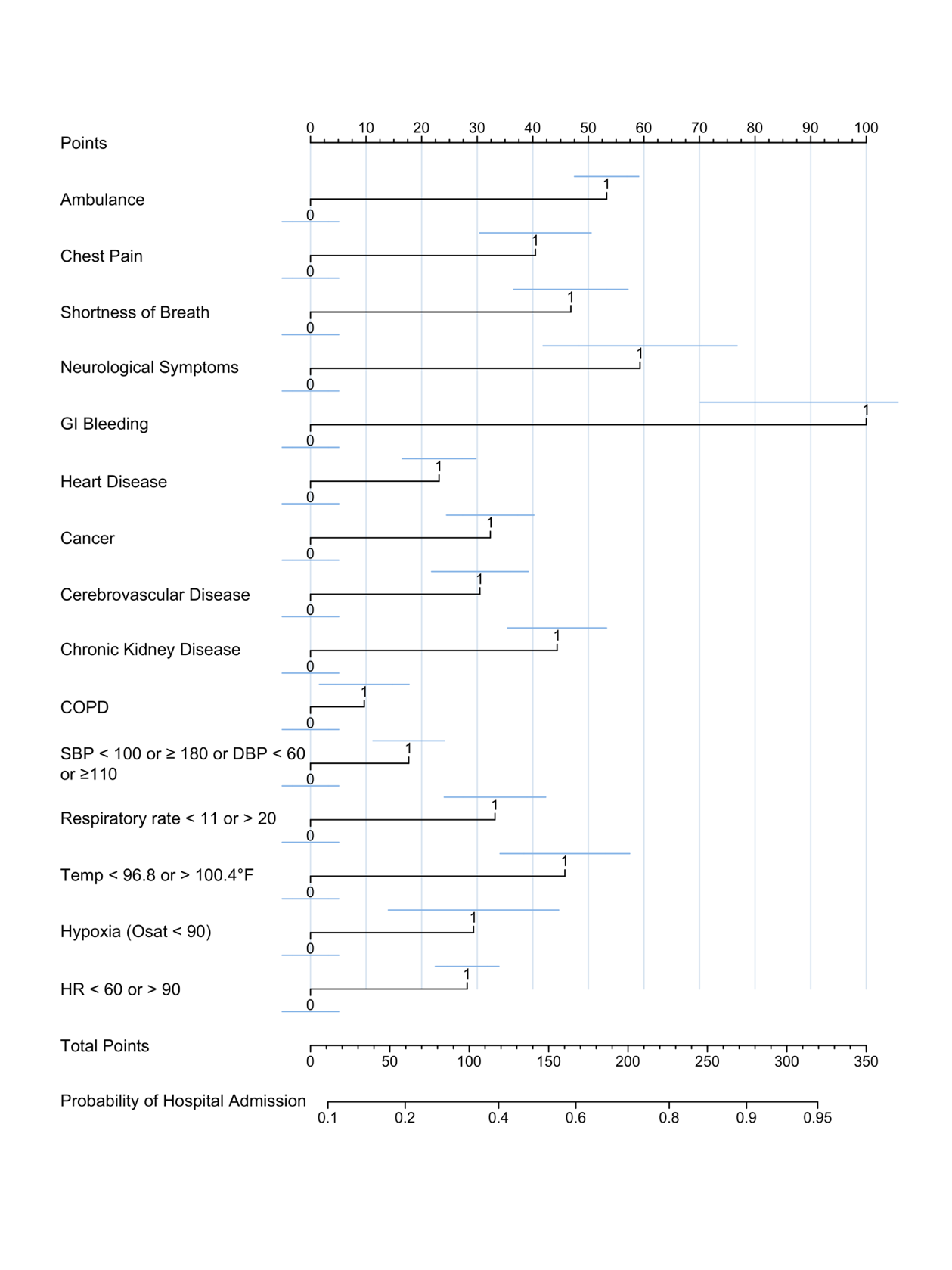
**

To use this nomogram, locate each patient's clinical variable on the respective rows and draw a vertical line up to the "Points" line to determine points for each factor. Sum all points to obtain a "Total Points" value, then draw a vertical line down from this total to the "Probability of Hospital Admission" line to estimate the patient's admission probability. For example, a patient arriving by ambulance (62 points) with chest pain (38 points) and hypoxia (50 points) would total 150 points, corresponding to an approximately 48% probability of admission.
